# Investigation of differences in xenophobia, prosociality level, and sociodemographic characteristics in nursing students

**DOI:** 10.1002/brb3.3277

**Published:** 2023-10-11

**Authors:** Tuba Korkmaz Aslan, Hacer Sönmezer Öcal

**Affiliations:** ^1^ Department of Nursing Necmettin Erbakan University Seydişehir Kamil Akkanat Faculty of Health Sciences Seydişehir Turkey; ^2^ Department of Nursing Karamanoglu Mehmetbey University Faculty of Health Sciences Karaman Turkey

**Keywords:** nursing students, prosocial behavior, xenophobia

## Abstract

**Aims:**

This study aimed to examine differences in xenophobia, prosocial behavior tendency, and sociodemographic characteristics among nursing students.

**Materials & Methods:**

The participants were 227 nursing students (29.1% male, 70.9% female) attending the nursing department in the spring semester of the 2019–2020 academic year. We used a personal identification form, xenophobia scale, and prosocial behavior tendency scale to collect the data.

**Results:**

Among the participants, 24.7% were in first year, 30.4% were in second year, 21.6% were in third year, and 23.3% were in fourth year. Female students had higher prosocial behavior disposition, altruistic, and submissive scores than their male counterparts. The second‐year students’ prosocial behavior scores were higher than those of the third‐year students. The male students’ hatred, humiliation, and xenophobia scores were higher than those of the female students.

**Discussion:**

The xenophobia scores were higher in those with two living parents and lower in those whose mothers had secondary education. We used the Kolmogorov–Smirnov test, Shapiro–Wilk test, and graphical assessments to test the quantitative data's compliance with normal distribution. We also employed the Student's *t*‐test and one‐way analysis of variance for data showing a normal distribution and Mann–Whitney *U* and Kruskal–Wallis tests for data showing non‐normal distribution. To evaluate the relationships among variables, we used the Pearson correlation analysis for normally distributed variables and Spearman's correlation analysis for non‐normally distributed variables.

**Conclusion:**

Women's prosocial behavior tendency total score was higher than men's, and men's xenophobia total score was higher than women's. There was a weak negative correlation between the positive behavior tendency and xenophobia total scores.

## Abstract 2

This study describes the differentiation of xenophobia and prosociality status of nursing students according to their sociodemographic characteristics.

## 1 BACKGROUND

Although xenophobia is becoming increasingly relevant in today's political climate, we know little about the impact of xenophobia on health. For the sake of a healthy society, students who will become health professionals should receive training in universal standards to exhibit positive social behaviors and provide holistic health services without discriminating against foreign national patients.

Xenophobia is an irrational fear of others (Lewicki, 2018) that arises in people who adopt an egocentric worldview and do not want to share what they have with others, and this sentiment commonly targets newcomers to a place (Akar & Erdoğdu, 2019; Mora, 2009; Yakushko, 2018). Although there is no exact numerical value to gauge xenophobia, it is widespread around the world and among health professionals (Selvarajah et al., 2022).

Migration is the movement of a population defined as the displacement of a person or group across national borders or within a country (TMA, 2016). Due to war, natural disasters, or political or financial reasons, people are forced to leave the places where they were born and have lived. Therefore, the concept of migration includes people and groups that must relocate, such as asylum seekers, refugees, economic migrants, and people relocating for other reasons (Akar & Erdoğdu, 2019; TMA, 2016). One of these groups is the Syrians living in Turkey. Internal turmoil, conflict, and war in Syria since 2010 have affected millions of people and caused significant migration movements toward Turkey. Refugees make up about 3% of Turkey's population (Ekici & Tuncel, 2015). Therefore, how Turkish health personnel communicate with foreigners and asylum seekers is important for ensuring the delivery of beneficial health services. It is thought that the behaviors and attitudes of health personnel toward foreigners and asylum seekers will affect both patient satisfaction and the quality of the service provided (Ciby & Raya, 2014; Dinc & Ertugrul, 2018). There is also a great need for the nursing workforce in various countries to encourage the migration of nursing professionals, which became especially salient during the pandemic period. In addition to the gap in the literature on xenophobia regarding the thoughts of nursing students, another rationale for this study is that the great need for growth in the nursing workforce brings xenophobia along with it (De Vries et al., 2023; Murphy et al., 2022; Stokes & Iskander, 2021).

Prosocial behavior is behavior that may benefit the individual or group, and the individual exhibits this behavior voluntarily and without pressure (Carlo et al., 2003; Yang et al., 2019). According to another definition, prosocial behavior represents a broad category of behavior that is generally beneficial to other people (Penner et al., 2005; Pfattheicher et al., 2022). As the relevant literature shows, authors have used the concepts of positive social behavior and prosocial behavior in the same sense (Çalık et al., 2009). The assumption is that people with a high propensity for positive social behavior are less likely to discriminate or have xenophobic thoughts. However, research has evinced that 97% of nurses working with foreign patients experience problems and difficulties, such as communication, language, cultural differences, and prejudice, during health service delivery (Cevik, 2018; Naami et al., 2020).

## 2 RATIONALE/JUSTIFICATION

Although xenophobia is becoming increasingly relevant in today's political climate, we know little about its impact on health. Given its many negative effects on individual and community health, xenophobia requires more public health attention. To ensure a healthy society, students who will become health professionals should receive education in human rights and universal standards to help them exhibit positive social behaviors and provide holistic health services without discriminating against foreign national patients.

The aim of this study was to examine the differentiation status of nursing students according to xenophobia, prosociality level, and sociodemographic characteristics. In this article, we describe the differentiation of xenophobia and prosociality status of nursing students according to their sociodemographic characteristics.

Accordingly, we formulated the following research questions:

1. Is there a difference in the prosociality status of student nurses according to their sociodemographic characteristics, such as age and gender?
2. Is there a difference in the xenophobia status of student nurses according to sociodemographic characteristics, such as the age and gender of student nurses?
3. Is there a relationship between the student nurses’ prosociality and xenophobia?

## 3 MATERIALS AND METHODS

### 3.1 Type of research

This was descriptive and correlational research.

### 3.2 Population and sample

In 2019, 677,042 people migrated to Turkey, representing an increase of 17.2% compared to the previous year (TÜİK, 2019). Turkey is among the countries that receive immigration in terms of geographical location. We determined that the population of our research would be two groups of nursing students from two diverse provinces of the Central Anatolia Region with easy access in order to ensure student diversity—namely, Karaman and Konya, which attract students from every region of Turkey.

The population of the study consisted of 525 nursing students enrolled in two universities. In line with previous studies that examined similar dependent variables, we calculated the sample size using the sample calculation formula in the groups, the population of which was known, and this yielded 222 students for the sample of the study (Sümbüloğlu & Sümbüloğlu, 2019). We reached out to these students by sending the questionnaire form link via the schools’ WhatsApp groups. Ultimately, we formed a sample group with 227 students who agreed to participate in the research.

### 3.3 Data collection technique and tools

During the pandemic, we contacted participants residing in many different cities online and collected the data online. We employed the survey program to prepare the electronic questionnaire, and we sent it to 227 students who agreed to participate in the research. In the field of health, nurses are the first to meet and communicate with patients. To determine the attitudes and approaches of nursing students in performing their duties and providing services to foreigners, we collected data online via a self‐diagnosis form, xenophobia scale, and prosocial behavior tendency scale.

### 3.4 Self‐diagnosis form

The self‐diagnosis form consisted of 13 questions in line with the literature (Barutçu & Zengin, 2018; Karataş & Güzel, 2020; Sünbül & Güçray, 2016; Tosun & Sinan, 2020).

### 3.5 Xenophobia scale

To determine the participants’ attitudes toward foreigners, we applied (Van Der Veer et al., 2011) 18‐item scale and followed Bozdağ and Kocatürk (2017) validity and reliability study in Turkey. The 18 items concerned asylum seekers, and the responses ranged from 1 = “I totally disagree” to 5 = “I totally agree.” In the evaluation, we reversed and scored the 11th and 7th items. On the scale, participants could score between 18 and 90 (Bozdağ & Kocatürk, 2017). Cronbach's alpha value of the xenophobia scale was .87 in the first application and .86 in the second stage, according to the confirmatory factor analysis. A high score indicated a high level of xenophobia. Cronbach's alpha value of the scale was .938.

### 3.6 Positive social behavior tendency

Carlo and Randall (2002) used the positive social behavior tendency scale to measure individuals’ prosocial behavior tendencies. Kumru et al. (2004) were responsible for the Turkish adaptation of the scale. Each item is in the form of a five‐point Likert scale from 1 = “It does not define me at all” to 5 = “Describes me very well.” 4, 10, 16, 20, 23. Articles are reverse‐scored. The scale consists of six subscales that are scored separately: Public OSD (“I can best help others when someone is watching me”), Emotional OSD, Altruistic OSD, Urgent OSD, Submissive OSD, and Covert OSD. Cronbach's alpha internal consistency coefficients of the scale for this study were .65 for the public prosocial behavior sub‐dimension, .63 for the emotional prosocial behavior sub‐dimension, .62 for the altruistic positive social behavior sub‐dimension, .43 for the positive social behavior in emergency situations sub‐dimension, .43 for the submissive positive social behavior sub‐dimension, .74 for the positive social behavior sub‐dimension, and .67 for the private positive social behavior sub‐dimension (Kumru et al., 2004, 2012). In this study, we applied a 23‐item questionnaire, where a high score on the scale revealed the individual's tendency to exhibit high prosocial behavior.

### 3.7 Data collection

The data collection for the study ran from 2 to May, 20 2021. We distributed the link to the survey to participants via social media accounts, email addresses, and phone numbers. To ensure that each participant filled in the questionnaire only once, we activated the “over one IP” and “one‐time fill” buttons on the page.

### 3.8 Variables

The continuous variables were the xenophobia and prosocial behavior tendency scale scores, and the categorical variables were sociodemographic characteristics (age, gender, province of education, income level, and place of residence).

### 3.9 Ethical dimension of the study

The Ethics Committee of Health Sciences Scientific Research of the researchers’ university granted approval for the study (April 7, 2021, Decision Number 26). Institutional permission was obtained. Before starting the data collection process, we apprised the participants of the purpose and scope of the research and obtained their online consent indicating their agreement to participate in the research. We also obtained scale usage permissions via email.

### 3.10 Limitations of the research

The limitation of the research lay in the collection of data during the pandemic and online. The population consisted of student nurses from provinces with easy access to all regions of Turkey. Due to the pandemic, there were many restrictions and even complete closures during the data collection phase, and the participants filled out the questionnaire with their families. The participants conveyed their thoughts in the questionnaire form in an environment where they could express their common culture and similar prejudices more easily with their families in different regions.

### 3.11 Statistical evaluation of the data

For the statistical analysis, we used the Number Cruncher Statistical System. To evaluate the data, we employed descriptive statistical methods (mean, standard deviation, median, frequency, ratio, minimum, maximum). We tested the compliance of the quantitative data with normal distribution via the Kolmogorov–Smirnov test, Shapiro–Wilk test, and graphical assessments. We adopted the Student's *t*‐test to compare quantitative data with the normal distribution between the two groups, and we applied the Mann–Whitney *U* test to compare variables that did not show a normal distribution. Gender presented a normal distribution in the Student's *t‐*test. We performed a one‐way analysis of variance (ANOVA) to compare three or more normally distributed groups, the Bonferroni test for pairwise comparisons, the Kruskal–Wallis test to compare three or more groups that did not show a normal distribution, and the Bonferroni–Dunn test for pairwise comparisons. Moreover, we employed the Pearson correlation analysis for normally distributed variables and Spearman's correlation analysis for non‐normally distributed variables to evaluate the relationships between variables. A significance of a minimum of *p* < .05 was acceptable.

## 4 RESULTS

The sample comprised 227 participants with a mean age of 21.06 ± 2.44 years, and 29.1% (*n* = 66) of the participants were male and 70.9% (*n* = 161) were female. Of the students participating in the study, 24.7% (*n* = 56) were in first year, 30.4% (*n* = 69) in second year, 21.6% (*n* = 49) in third year, and 23.3% (*n* = 53) in fourth year. In addition, 60.4% (*n* = 137) of the students were studying in Karaman and 43.6% (*n* = 99) of them lived in dormitories. Furthermore, in the case of 94.3% (*n* = 214) of the students, both their mother and father were alive, 5.7% (*n* = 13) of the students lived separately from their parents, and 20.7% (*n* = 47) had four or more siblings. Lastly, 88.1% (*n* = 200) of the participants’ mothers were not working, and 27.8% (*n* = 63) of the participants had an income that was not commensurate with their expenses (Table 1).

**TABLE 1:** Distribution of demographic and family characteristics.

**Gender;** *n* (%)	Male	66 (29.1)
	Female	161 (70.9)
**Age (year)**	*Avg*. ± *Ss*	21.06 ± 2.44
	*Median (min*–*max)*	21 (18–41)
**Class;** *n* (%)	First year	56 (24.7)
	Second year	69 (30.4)
	Third year	49 (21.6)
	Fourth year	53 (23.3)
**City where the university is studied;** *n* (%)	Konya	90 (39.6)
	Karaman	137 (60.4)
**Place of residence;** *n* (%)	Dormitory	99 (43.6)
	Student house	18 (7.9)
	With family	106 (46.7)
	Other	4 (1.8)
**Are the parents alive?;** *n* (%)	Alive	214 (94.3)
	Only mother is alive	11 (4.8)
	Only father is alive	2 (0.9)
**Parental union;** *n* (%)	Lives together	204 (89.9)
	Separated	13 (5.7)
	Other	10 (4.4)
**Number of siblings excluding yourself;** *n* (%)	1 Sibling	57 (25.1)
	2 Siblings	90 (39.6)
	3 Siblings	33 (14.5)
	≥4 siblings	47 (20.7)
**Father's education status;** *n* (%)	Primary education	99 (43.6)
	Secondary education	86 (37.9)
	University	42 (18.5)
**Mother's education status;** *n* (%)	Primary education	162 (71.4)
	Secondary education	52 (22.9)
	University	13 (5.7)
**Mother's occupation;** *n* (%)	Unemployed	200 (88.1)
	Employed	23 (10.1)
	Retired	4 (1.8)
**Income and expense status;** *n* (%)	Income is more than expenses	30 (13.2)
	Income is equal to expenses	134 (59.0)
	Income is less than expenses	63 (27.8)

The averages of the dimensions of the prosocial behavior tendency scale were public (1.59 ± 0.72), emotional (3.75 ± 0.79), altruistic (3.78 ± 0.84), in emergency situations (3.48 ± 0.83), obedient (3.71 ± 0.95), and confidential (4.03 ± 0.78). The total scores for the scale ranged from 2.3 to 4.22, with an average of 3.40 ± 0.39.

Upon examining Cronbach's alpha values, which show the internal consistency of the scale, it became clear that each sub‐dimension had a different value, and the highest was .801 and the lowest was .542. Cronbach's alpha value for the total of the prosocial behavior tendency scale was .805, showing the high reliability of our scale (Table 2).

**TABLE 2:** Distribution of positive social behavior tendency scale scores and internal consistency values.

Prosocial behavior tendency scale	Number of articles	Median (min–max)	Avg. ± Ss	Cronbach's alpha
**Public**	4	1.25 (1–5)	1.59 ± 0.72	.708
**Emotional**	4	3.75 (1.75–5)	3.75 ± 0.79	.657
**Altruist**	5	3.80 (1–5)	3.78 ± 0.84	.670
**Emergency**	3	3.33 (1–5)	3.48 ± 0.83	.542
**Submissive**	2	4 (1–5)	3.71 ± 0.95	.801
**Private**	5	4.20 (1.8–5)	4.03 ± 0.78	.747
**Total**	23	3.43 (2.3–4.22)	3.40 ± 0.39	.805

The mean score of the hate sub‐dimension of the xenophobia scale was 15.16 ± 5.90, the mean score of the fear sub‐dimension was 23.21 ± 5.86, the mean score of the humiliation sub‐dimension was 9.19 ± 3.67, and the total score of the xenophobia scale ranged from 20 to 87, with an average of 47.56 ± 13.80.

Cronbach's alpha values showing the internal consistency of the xenophobia scale were .893 for the hate sub‐dimension, .867 for the fear sub‐dimension, and .868 for the humiliation sub‐dimension. Cronbach's alpha value for the total xenophobia scale was .938, showing the high reliability of our scale (Table 3).

**TABLE 3:** Distribution of xenophobia scale scores and internal consistency values.

Xenophobia scale	Number of articles	Median (min–max)	Avg. ± Ss	Cronbach's alpha
**Hate**	7	14 (7–35)	15.16 ± 5.90	.893
**Fear**	7	23 (8–35)	23.21 ± 5.86	.867
**Humiliation**	4	8 (4–20)	9.19 ± 3.67	.868
**Total**	18	45 (20–87)	47.56 ± 13.80	.938

We found a statistically significant but very weak relationship (as age increases, the altruism score decreases) between the participants’ ages and the altruistic sub‐dimension score of the prosocial behavior tendency scale. There was also a statistically significant gender difference between the prosocial behavior tendency total score and altruistic and submissive scores (sub‐dimensions of prosocial behavior tendency). The girls’ scores were higher than those of the boys (*p* < .05). There was a statistically significant difference between altruistic scores by class level. Paired comparisons showed that the first‐year students’ altruistic scores were higher than those of the fourth‐year students (*p* < .05). We also identified a statistically significant difference between submissive scores by class level. Paired comparisons revealed that the submissive scores of second‐year students were higher than those of third‐year students (*p* < .05). There was also a statistically significant difference between the prosocial behavior tendency total scores by class level. Paired comparisons evinced that the second‐year students’ prosocial behavior tendency total scores were higher than those of the third‐year students (*p* < .05). In addition, there was a statistically significant difference between altruistic scores by place of residence. Pairwise comparisons aimed at determining which group created the difference indicated that the altruistic scores of those living in dormitories and with their families were higher than those of participants living in student houses (*p* < .05).

Furthermore, there was a statistically significant difference between submissive scores according to the father's educational status. Pairwise comparisons showed that the submissive scores of students whose fathers were primary school graduates were higher than those whose fathers were university graduates (*p* < .05). There was also a statistically significant difference between submissive scores according to the mother's educational status. Paired comparisons revealed that the submissive scores of students whose mothers were primary school graduates were higher than those of students whose mothers were secondary school graduates (*p* < .05). There was a statistically significant difference between private scores according to income status. Paired comparisons revealed that the private scores of students whose income was less than their expenses were higher than those whose income was equivalent to their expenses (*p* < .01; Table 4).

**TABLE 4:** Distribution of positive social behavior tendency scale scores by demographic characteristics.

			Prosocial behavior tendency scale						
			Public	Emotional	Altruist	Emergency	Submissive	Private	Total
**Age (year)**	**r**		.065^a^	−.027^b^	−.235^b^	.078^b^	.067^b^	−.006^b^	−.033^b^
	**p**		.327	.686	**.001** ^**^	.242	.316	.924	.619
**Gender**	**Female (n = 161)**	Avg. ± Ss	1.50 ± 0.66	3.80 ± 0.82	3.87 ± 0.81	3.50 ± 0.83	3.79 ± 0.96	4.10 ± 0.76	3.44 ± 0.40
		Median (min–max)	1.3 (1–5)	3.8 (1.8–5)	4 (1–5)	3.3 (1–5)	4 (1–5)	4.2 (2.2–5)	3.5 (2.3–4.2)
	**Male (n = 66)**	Avg. ± Ss	1.82 ± 0.80	3.62 ± 0.70	3.59 ± 0.91	3.45 ± 0.84	3.52 ± 0.91	3.88 ± 0.81	3.33 ± 0.35
		Median (min–max)	1.5 (1–4.3)	3.6 (2–5)	3.8 (1–5)	3.3 (1–5)	3.5 (1–5)	4 (1.8–5)	3.3 (2.6–4)
		**p**	**0.001** ^c**^	0.116^d^	**0.026** ^d*^	0.672^d^	**0.048** ^d*^	0.058^d^	**0.046** ^d*^
**Year**	**First year (n = 56)**	Avg. ± Ss	1.48 ± 0.60	3.75 ± 0.87	3.96 ± 0.80	3.46 ± 0.87	3.55 ± 1.07	4.11 ± 0.72	3.42 ± 0.41
		Median (min–max)	1.3 (1–3)	4 (1.8–5)	4.1 (1.6–5)	3.3 (1–5)	3.5 (1–5)	4.2 (2.2–5)	3.5 (2.3–4.2)
	**Second year (n = 69)**	Avg. ± Ss	1.54 ± 0.71	3.94 ± 0.75	3.85 ± 0.84	3.63 ± 0.83	3.99 ± 0.82	4.14 ± 0.78	3.51 ± 0.36
		Median (min–max)	1.3 (1–5)	4 (2–5)	4 (1–5)	3.7 (1–5)	4 (1–5)	4.2 (1.8–5)	3.5 (2.6–4.2)
	**Third year (n = 49)**	Avg. ± Ss	1.55 ± 0.62	3.62 ± 0.73	3.80 ± 0.73	3.28 ± 0.76	3.50 ± 0.91	3.97 ± 0.75	3.32 ± 0.40
		Median (min–max)	1.5 (1–3.5)	3.5 (2.3–5)	3.8 (2.4–5)	3.3 (1.7–5)	3.5 (2–5)	4.2 (2.2–5)	3.3 (2.5–4)
	**Fourth year (n = 53)**	Avg. ± Ss	1.81 ± 0.88	3.61 ± 0.79	3.51 ± 0.96	3.51 ± 0.83	3.71 ± 0.94	3.90 ± 0.85	3.33 ± 0.38
		Median (min–max)	1.5 (1–4.3)	3.5 (1.8–5)	3.8 (1–5)	3.7 (1.3–5)	4 (1–5)	4 (2.2–5)	3.3 (2.5–4.1)
		**P**	0.361^e^	0.071^f^	**0.039** ^f*^	0.153^f^	**0.020** ^f*^	0.297^f^	**0.023** ^f*^
**Place of residence**	**Dormitory (n = 99)**	Avg. ± Ss	1.62 ± 0.71	3.78 ± 0.77	3.83 ± 0.78	3.44 ± 0.78	3.75 ± 0.92	3.98 ± 0.79	3.41 ± 0.38
		Median (min–max)	1.5 (1–5)	3.8 (2–5)	3.8 (1–5)	3.3 (1.7–5)	4 (1–5)	4 (1.8–5)	3.4 (2.6–4.1)
	**Student house (n** **=** **18)**	Avg. ± Ss	2.00 ± 1.01	3.78 ± 0.79	3.21 ± 0.83	3.59 ± 1.06	3.44 ± 0.92	3.96 ± 0.91	3.33 ± 0.39
		Median (min–max)	1.6 (1–4.3)	3.8 (2.5–5)	3.1 (1.8–4.8)	3.5 (1–5)	3.8 (1–5)	4.1 (2.4–5)	3.4 (2.6–3.9)
	**Homestay (n = 106)**	Avg. ± Ss	1.51 ± 0.65	3.72 ± 0.81	3.83 ± 0.87	3.50 ± 0.86	3.74 ± 0.97	4.11 ± 0.74	3.42 ± 0.40
		Median (min–max)	1.3 (1–5)	3.8 (1.8–5)	4 (1–5)	3.7 (1–5)	4 (1–5)	4.2 (2.2–5)	3.4 (2.3–4.2)
	**Other** ^g^ **(n = 4)**	Avg. ± Ss	1.13 ± 0.25	3.50 ± 1.17	4.25 ± 1.04	3.67 ± 0.27	3.13 ± 1.18	3.95 ± 0.87	3.34 ± 0.41
		Median (min–max)	1 (1–1.5)	4 (1.8–4.3)	4.6 (2.8–5)	3.7 (3.3–4)	3.5 (1.5–4)	3.9 (3.2–4.8)	3.4 (2.8–3.7)
		**P**	0.126^e^	0.888^f^	**0.011** ^f*^	0.736^f^	0.437^f^	0.460^f^	0.676^f^
**Father's educational status**	**Primary education (n = 99)**	Avg. ± Ss	1.67 ± 0.79	3.81 ± 0.76	3.71 ± 0.91	3.53 ± 0.80	3.87 ± 0.91	4.05 ± 0.77	3.44 ± 0.39
		Median (min–max)	1.5 (1–5)	3.8 (2.3–5)	3.8 (1–5)	3.7 (1.7–5)	4 (1–5)	4.2 (1.8–5)	3.4 (2.6–4.2)
	**Secondary education (n = 86)**	Avg. ± Ss	1.57 ± 0.73	3.77 ± 0.86	3.86 ± 0.82	3.49 ± 0.88	3.66 ± 0.95	4.05 ± 0.83	3.42 ± 0.40
		Median (min–max)	1.3 (1–4.3)	4 (1.8–5)	3.9 (1.8–5)	3.3 (1–5)	4 (1–5)	4.2 (2.2–5)	3.4 (2.5–4.1)
	**University (n = 42)**	Avg. ± Ss	1.43 ± 0.46	3.54 ± 0.71	3.84 ± 0.75	3.37 ± 0.80	3.43 ± 0.98	3.98 ± 0.70	3.30 ± 0.37
		Median (min–max)	1.3 (1–2.5)	3.5 (1.8–5)	3.8 (1.8–5)	3.3 (1–5)	3.5 (1–5)	4 (2.4–5)	3.3 (2.3–3.8)
		**P**	0.421^e^	0.152^f^	0.443^f^	0.585^f^	**0.035** ^f*^	0.872^f^	0.150^f^
**Mother's educational level**	**Primary education (n = 162)**	Avg. ± Ss	1.59 ± 0.72	3.81 ± 0.80	3.79 ± 0.84	3.55 ± 0.84	3.80 ± 0.96	4.09 ± 0.76	3.45 ± 0.39
		Median (min–max)	1.3 (1–5)	3.8 (1.8–5)	4 (1–5)	3.7 (1–5)	4 (1–5)	4.2 (1.8–5)	3.4 (2.3–4.2)
	**Secondary education (n = 52)**	Avg. ± Ss	1.62 ± 0.75	3.61 ± 0.78	3.72 ± 0.91	3.33 ± 0.84	3.50 ± 0.96	3.96 ± 0.81	3.32 ± 0.37
		Median (min–max)	1.5 (1–4.3)	3.5 (2–5)	3.7 (1.6–5)	3.3 (1.7–5)	3.5 (1–5)	4.1 (2.2–5)	3.3 (2.5–4)
	**University (n = 13)**	Avg. ± Ss	1.48 ± 0.58	3.56 ± 0.63	4.03 ± 0.72	3.31 ± 0.54	3.42 ± 0.53	3.71 ± 0.89	3.29 ± 0.33
		Median (min–max)	1.3 (1–2.5)	3.8 (2.5–4.5)	4 (3–5)	3.3 (2.3–4)	3.5 (2.5–4)	3.6 (2.2–5)	3.3 (2.7–3.7)
		**P**	0.858^e^	0.153^e^	0.560^e^	0.137^e^	**0.032** ^e*^	0.213^e^	0.054^e^
**Level of income**	**Income is more than expenses (n =** **30)**	Avg. ± Ss	1.72 ± 0.79	3.87 ± 0.80	3.61 ± 0.88	3.78 ± 0.69	3.68 ± 0.87	4.01 ± 0.70	3.44 ± 0.38
		Median (min–max)	1.5 (1–4.3)	4 (2.3–5)	3.6 (1.8–5)	3.8 (2.3–5)	3.5 (2–5)	4 (2.6–5)	3.5 (2.6–4.2)
	**Income is equal to expenses (n = 134)**	Avg. ± Ss	1.61 ± 0.69	3.70 ± 0.73	3.78 ± 0.80	3.43 ± 0.82	3.67 ± 0.92	3.91 ± 0.81	3.36 ± 0.38
		Median (min–max)	1.5 (1–3.5)	3.8 (1.8–5)	3.8 (1.6–5)	3.3 (1–5)	4 (1–5)	4 (1.8–5)	3.4 (2.3–4.2)
	**Income is less than expenses (n = 63)**	Avg. ± Ss	1.48 ± 0.73	3.79 ± 0.92	3.89 ± 0.92	3.47 ± 0.90	3.80 ± 1.06	4.31 ± 0.69	3.48 ± 0.41
		Median (min–max)	1.3 (1–5)	3.8 (1.8–5)	4.2 (1–5)	3.3 (1.3–5)	4 (1–5)	4.6 (2.4–5)	3.5 (2.5–4.1)
		**p**	0.136^e^	0.530^f^	0.313^f^	c0.114^f^	0.663^f^	**0.003** ^f**^	0.120^f^

^b^ *r*: Pearson correlation coefficient.

^a^ *r*: Spearman's correlation coefficient.

^c^ Mann–Whitney *U* test.

^d^ Student *t*‐test.

^e^ Kruskal–Wallis test.

^f^ One‐way ANOVA test.

^g^ As the number of people was small, it was not included in the statistical evaluation.

* *p* < .05.

** *p* < .01.

We found a statistically significant difference among the hate, fear, and humiliation scores according to gender (*p* < .01). Men's hate, fear, and humiliation scores were higher than those of women. There was also a statistically significant difference among xenophobia total scores according to gender (*p* < .01). Men's overall scores were higher than women's. We identified a statistically significant difference between hate scores and xenophobia total scores according to the parents’ health status. Those with two living parents had higher hate scores than those with only a surviving mother (*p* < .05). There was also a statistically significant difference between hate scores according to the mother's educational status. Paired comparisons revealed that the hate scores of those whose mothers were secondary school graduates were higher than those whose mothers were primary school graduates (*p* < .05). We uncovered a statistically significant difference between the humiliation and xenophobia scores according to the mother's educational status. Pairwise comparisons indicated that the humiliation scores of students whose mothers were university and secondary school graduates were higher than those of students whose mothers were primary school graduates (*p* < .05). We found a statistically significant difference among fear scores according to income level. Paired comparisons revealed that the fear scores of those whose income was less than their expenses were significantly higher than those whose income was equal to their expenses (*p* < .05; Table 5).

**TABLE 5:** Distribution of xenophobia scale scores by demographic characteristics.

			Xenophobia scale			
			Hate	Fear	Humiliation	Total
**Age (year)**	**r**		.029^a^	−.023^a^	−.002^b^	−.005
	**p**		**.663**	**.733**	**.980**	**.945**
**Gender**	**Female (n = 161)**	Avg. ± Ss	14.28 ± 5.13	22.41 ± 5.44	8.77 ± 3.50	45.46 ± 12.58
		Median (min–max)	14 (7–33)	22 (8–35)	8 (4–20)	44 (20–82)
	**Male (n = 66)**	Avg. ± Ss	17.32 ± 7.04	25.15 ± 6.42	10.23 ± 3.91	52.70 ± 15.34
		Median (min–max)	16 (8–35)	25.5 (12–35)	10 (4–20)	52.5 (26–87)
		**p**	**0.002** ^c**^	**0.001** ^c**^	**0.005** ^d**^	**0.001** ^c**^
**Parental health status**	**Parents are alive (n = 214)**	Avg. ± Ss	15.24 ± 5.85	23.29 ± 5.93	9.25 ± 3.67	47.78 ± 13.81
		*Median (min–max)*	14 (7–35)	23 (8–35)	8.5 (4–20)	46 (20–87)
	**Only mother is alive (*n* = 11)**	*Avg*. ± *Ss*	11.82 ± 3.57	20.73 ± 3.77	7.64 ± 2.50	40.18 ± 7.68
		*Median (min–max)*	11 (8–19)	21 (14–27)	8 (4–12)	39 (30–58)
	**Only father is alive** ^e^ **(*n* = 2)**	*Avg*. ± *Ss*	25.00 ± 11.31	28.00 ± 5.66	12.00 ± 8.49	65.00 ± 25.46
		*Median (min–max)*	25 (17–33)	28 (24–32)	12 (6–18)	65 (47–83)
		**p**	**0.037** ^d*^	0.156^d^	0.187^d^	**0.044** ^d*^
**Mother's educational level**	**Primary education (n = 162)**	Avg. ± Ss	14.52 ± 5.75	22.86 ± 5.92	8.71 ± 3.73	46.09 ± 13.68
		Median (min–max)	13 (7–34)	23 (8–35)	8 (4–20)	44 (20–87)
	**Secondary education (n = 52)**	Avg. ± Ss	16.73 ± 6.06	23.88 ± 5.76	10.12 ± 3.22	50.73 ± 13.62
		Median (min–max)	15 (7–35)	22.5 (12–35)	10 (4–20)	47.5 (30–82)
	**University (n = 13)**	Avg. ± Ss	16.92 ± 5.92	24.77 ± 5.45	11.54 ± 3.33	53.23 ± 13.47
		Median (min–max)	16 (10–28)	25 (15–34)	10 (8–20)	51 (33–79)
		**p**	**0.012** ^f*^	0.320^f^	**0.001** ^f**^	**0.030** ^f*^
**Level of income**	**Income is more than expenses (n = 30)**	Avg. ± Ss	16.13 ± 6.37	24.60 ± 6.46	10.30 ± 3.48	51.03 ± 14.96
		Median (min–max)	15 (7–35)	24.5 (8–35)	9.5 (5–20)	51 (20–82)
	**Income is equal to expenses (n = 134)**	Avg. ± Ss	14.97 ± 6.03	22.22 ± 5.83	9.01 ± 3.67	46.21 ± 14.02
		Median (min–max)	14 (7–34)	21 (10–35)	8 (4–20)	44 (22–87)
	**Income is less than expenses (n = 63)**	Avg. ± Ss	15.11 ± 5.41	24.63 ± 5.27	9.05 ± 3.75	48.79 ± 12.53
		Median (min–max)	14 (9–30)	24 (11–35)	8 (4–18)	46 (24–79)
		**p**	0.621^g^	**0.010** ^g*^	0.158^f^	0.159^g^

^a^ *r*: Pearson correlation coefficient.

^b^ *r*: Spearman's correlation coefficient.

^c^ Student *t*‐test.

^d^ Mann–Whitney *U* test.

^e^ As the number of people was small, it was not included in the statistical evaluation.

^f^ Kruskal–Wallis test.

^g^ One‐way ANOVA test.

* *p* < .05.

** *p* .01.

There was a very weak positive correlation between the public score and the hate, humiliation, and xenophobia total scores (respectively, *r* = .178; *r* = .188; *r* = .159; *p* < .05). We identified a negative statistically significant very weak/weak correlation between the altruistic scores and the hate, fear, humiliation, and xenophobia total scores (respectively, *r* = −.276; *r* = −.139; *r* = −.194; *r* = −.238; *p* < .05).

There was a very weak negative, statistically significant correlation between the in emergency situations and hate scores (*r* = −.172; *p* < .05). There was a very weak negative, statistically significant correlation between the submissive scores and the hate and humiliation scores (respectively, *r* = −.202; *r* = −.154; *p* < .05). There was a statistically significant and very weak negative correlation between the private score and the hate and humiliation scores (respectively, *r* = −.158; *r* = −.143; *p* < .05). There was a statistically significant and very weak negative correlation between the positive behavior tendency total score and the hate and humiliation total scores (respectively, *r* = −.232, *r* = −.148; *p* < .05; Table 6).

**TABLE 6:** The relationship between positive behavior tendency scale and xenophobia scale scores.

		Prosocial behavior tendency scale						
Xenophobia scale		Public	Emotional	Altruist	Emergency	Submissive	Private	Total
**Hate**	***r***	.178^b^	−.082^a^	−.276^a^	−.172^a^	−.202^a^	.158^a^	−.232^a^
	***p***	***.007*** ^c^	*.221*	***.001*** ^c^	***.009*** ^c^	***.002*** ^c^	***.017*** ^d^	***.001*** ^c^
**Fear**	***r***	.059^b^	.079^a^	−.139^a^	.034^a^	.016^a^	.040^a^	.015^a^
	***p***	*.376*	*.236*	***.037*** ^d^	*.610*	*.810*	*.545*	*.827*
**Humiliation**	***r***	.188^b^	.021^b^	−.194^b^	.067^a^	−.154^b^	−.143^b^	−.148^b^
	***p***	***.005*** ^c^	*.749*	***.003*** ^c^	*.315*	***.020*** ^d^	***.032*** ^d^	***.026*** ^d^
**Total**	***r***	.159^b^	.013^a^	−.238^a^	−.082^a^	−.113^a^	−.079^a^	−.125^a^
	***p***	***.017*** ^d^	*.849*	***.001*** ^c^	*.217*	***.090***	*.236*	***.060***

^a^ *r*: Pearson correlation coefficient.

^b^ *r*: Spearman's correlation coefficient.

^c^ *p* < 0.01.

^d^ *p* 0.05.

## 5 DISCUSSION

Now, we will discuss the results of this study in line with the research questions. Female students’ prosocial behavior tendency total score was higher than men's, and men's xenophobia total score was higher than women's. Health is a human right, and many people migrate to different countries. People's health status can change in a positive or negative direction at any time, especially when they start a new life in a different country, due to climatic conditions, economic conditions, and so on. The level of health status differs in relation to many factors. The nursing profession is of great importance in the care and treatment provided in healthcare services (Buheji & Buhaid, 2020; Namdar, 2019).

As in other countries, the increase in the number of foreigners due to globalization and world politics affects students to a great extent in Turkey. In this study, which examined differences in xenophobia, prosociality level, and sociodemographic characteristics among nursing students, we observed a difference between the prosocial behavior tendencies of female and male students. Women scored high on the altruistic and submissive positive behavior tendency sub‐dimensions, whereas men scored high on the public sub‐dimension. Aktaş and Güvenç (2006) found that men exhibited more public prosocial behavior, whereas women exhibited more covert and submissive prosocial behavior. In total, we found that the tendency of women to show prosocial behavior was high. This supports a previous study showing that women's prosocial behavior was high (Sünbül & Güçray, 2016). In Turkey, people perceived the nursing profession as a feminine profession until very recently, and they viewed the need for care and assistance as instinctive and thus associated it with a sister or a mother. This study shows us that a different attitude or prejudice does not change the approach of women toward people who need help.

Men's xenophobia total score was higher than that of women. By contrast, Güngör et al. (2021) found higher xenophobia scores in women. Similarly, Karataş and Güzel (2020) reported that among students enrolled in various departments at a university, men's levels of xenophobia were higher than women's. However, the increase in the number of asylum seekers, the prolongation of their stay, the recent economic and social problems, and the belief that refugees have deepened these problems, combined with the anxiety about the future of young university students especially, may have caused an increase in xenophobic thoughts about the number of refugees (Tosun & Sinan, 2020).

In this study, we found no significant differences between the prosociality and xenophobia scores of nursing students according to their class level. Other studies found that nurses with higher knowledge and education levels had more positive attitudes toward refugees (Lee et al., 2017; Tosun & Sinan, 2020).

Most nurses working in hospitals in Turkey have a secondary school education or an associate, undergraduate, or graduate degree. In this study, the participants’ class level supported the connection between their professional and practical approaches and their education level. Technological development and an increase in international travel or migration also reinforce the importance of the relationship between our attitudes and approaches to foreigners (Indelicato et al., 2022; Şendir et al., 2019).

Although some studies have reported that individuals with a low level of education may be less tolerant toward asylum seekers and develop negative attitudes toward them (Barutçu & Zengin, 2018; Campbell et al., 2018; Rousseau et al., 2017), in our study, as the mother's level of education decreased, the participant's xenophobia total score also decreased. This may be a result of being detached from the outside world and spending less time on social media as the level of education decreases.

The xenophobia levels of health education students may affect the care they give to patients (Yıldız et al., 2023). Researchers should, first, determine the levels of xenophobia and prosocial behavior in nursing students by conducting descriptive studies, and then the curriculum should include these issues, supported by randomized studies.

Another aim of the study was to determine the relationship between nursing students’ prosocial behavior levels and xenophobia. There was a negative correlation between the positive behavior tendency total scores and xenophobia total scores. No other research results highlighted a relationship between prosociality and xenophobia.

### 5.1 Relevance for clinical practice

In order for nursing candidates to develop positive attitudes, they need education about different cultures and human rights and support. This is necessary for future job satisfaction and quality of care. The curriculum must include such concepts to promote understanding of intercultural care and eliminate racism and stigmatization.

## 6 CONCLUSION AND RECOMMENDATIONS

The results of this study show that the prosociality level of women was higher than that of men, and the men's xenophobia scores were high. This suggests that gender has an effect on xenophobia and prosociality. We observed that the xenophobia score decreased as prosociality increased; thus, prosocial behavior in nursing students reduced xenophobia. If there are programs that improve the level of prosociality in nursing students, this will be beneficial in eliminating xenophobia. As the nursing oath states, “I will take care of my patients with the understanding and skills I have developed with the awareness of the responsibilities I have undertaken. Regardless of any race, belief, color, political or social situation, I will make every effort to protect life, alleviate suffering, and promote health.” All nursing students should receive training in combating xenophobia.

We argue that adding prosociality and xenophobia topics to the curriculum will improve nursing students’ prosocial behavior and reduce their prejudice against foreigners. We can deduce that nursing students and those working in the nursing profession should receive support with the necessary course curriculum or in‐service training to increase their positive prosocial behavior tendencies.

## AUTHOR CONTRIBUTIONS

**Tuba Korkmaz Aslan**: Conceptualization; data curation; formal analysis; funding acquisition; investigation; methodology; project administration; resources; software; supervision; validation; visualization; writing—original draft; writing—review and editing. **Hacer Sönmezer Öcal**: Data curation; formal analysis; funding acquisition; investigation; methodology; project administration; software; supervision; validation; visualization; writing—review and editing.

## CONFLICT OF INTEREST STATEMENT

The authors declare no conflicts of interest.

## FUNDING INFORMATION

This research did not receive any specific grant from funding agencies in the public, commercial, or not‐for‐profit sectors.

### PEER REVIEW

The peer review history for this article is available at https://publons.com/publon/10.1002/brb3.3277.

## INFORMED CONSENT

(For case reports) Online informed consent was obtained from the nursing student for publication and images.

## Data Availability

I declare the availability of data.
